# Sulforaphane Alleviates Zearalenone-Induced Oxidative Stress in Bovine Mammary Epithelial Cells

**DOI:** 10.3390/ani16111602

**Published:** 2026-05-25

**Authors:** Yurong Fu, Tingting Liu, Peng Peng, Xi Chen, Siwei Wang, Shuang Liang, Shaoqing Shi, Chuanqi Wang, Kun Wang

**Affiliations:** 1 Hebei Key Laboratory of Crop Cultivation Physiology and Green Production, Institute of Cereal and Oil Crops, Hebei Academy of Agriculture and Forestry Sciences, Shijiazhuang 050035, China; fuyurong2023@163.com (Y.F.); 15930885886@163.com (T.L.); pengp1994@163.com (P.P.); chenx202012@163.com (X.C.); wangsiwei0909@126.com (S.W.); wyoubbjmeefn@gmail.com (S.L.); shisq1991@163.com (S.S.); 2 Jilin Provincial Key Laboratory of Livestock and Poultry Feed and Feeding in the Northeastern Frigid Area, College of Animal Sciences, Jilin University, Changchun 130062, China

**Keywords:** sulforaphane, zearalenone, oxidative damage, Nrf2 signaling pathway, bovine mammary epithelial cells

## Abstract

Zearalenone (ZEA) is a common contaminant in crops and animal feed. However, research on the effects of ZEA on animal mammary tissue is relatively limited. Sulforaphane (SFN) is a naturally active compound mainly derived from cruciferous vegetables (such as broccoli), with significant antioxidant and cytoprotective effects. The purpose of this study is the effect of SFN on ZEA-induced toxicity in bovine mammary epithelial cells (MAC-T). By treating MAC-T cells with different concentrations of ZEA and SFN for 24 h, the results showed that different concentrations of ZEA (10, 20, 40, 60, 80, or 100 μM) could inhibit MAC-T cell viability. Treatment with SFN at concentrations of 1, 2.5, and 5 μM had no significant effect on cell viability. The results of combined treatment with 10 μM ZEA and 1, 2.5, or 5 μM SFN showed that SFN could significantly reverse the decrease in cell viability caused by ZEA; reduce the increase in lactate dehydrogenase (LDH) release, reactive oxygen species (ROS), and malondialdehyde (MDA) content induced by ZEA; and increase the levels of glutathione (GSH), superoxide dismutase (SOD), and mitochondrial membrane potential that were decreased by ZEA. SFN can significantly inhibit the upregulation of interleukin 6 (IL-6), tumor necrosis factor alpha (TNF-α), and interleukin 1 beta (IL-1β) induced by ZEA exposure and markedly reverse the increase in cell apoptosis rate caused by ZEA. Compared with the control group, the expression of genes nuclear factor erythroid 2–related factor 2 (*Nrf2*), heme oxygenase 1 (*HO-1*), NAD(P)H:quinone oxidoreductase 1 (*NQO1*), glutamate-cysteine ligase modifier subunit (*GCLM*), and glutathione peroxidase 1 (*GPX1*) was significantly reduced in the ZEA group, while the addition of SFN effectively increased the expression levels of these genes. Corresponding protein detection results were consistent with the trends in gene expression. This study demonstrated that SFN alleviates ZEA-induced damage to MAC-T cells by activating the Nrf2 pathway, providing a theoretical basis for the subsequent application of SFN in dairy farming to prevent and control breast health risks related to mycotoxins.

## Simple Summary

Mycotoxin contamination is widespread. Among these, the common mycotoxin zearalenone (ZEA) poses a potential risk to the health of dairy cows. In this study, we aimed to test whether a natural compound from broccoli, sulforaphane (SFN), can protect bovine mammary epithelial cells from ZEA-induced toxic damage. Our results show that SFN can effectively reduce cell apoptosis and inflammation, and activate the Nrf2 signaling pathway to alleviate oxidative stress. We conclude that SFN is a promising protective agent against ZEA toxicity. This finding provides a potential natural strategy to improve animal health and food safety, benefiting both the livestock industry and public health.

## 1. Introduction

Foods, feed, and grains are often co-contaminated by multiple mycotoxins [1]. Zearalenone (ZEA) is one of the common mycotoxins and is widely present in crops [2]. Feed contaminated with ZEA often causes animals to exhibit various adverse reactions. It is reported that ZEA and its metabolites cause various harms to humans and animals, including reproductive toxicity [3], cytotoxicity [4], hepatotoxicity [5] and nephrotoxicity [6], with their reproductive effects receiving widespread attention. ZEA exhibits dose-dependent effects: low doses of ZEA can induce cell proliferation and carcinogenesis [7,8], whereas high doses can lead to a series of stress and damage, as well as cell apoptosis [9,10]. Although many methods to prevent the production of mycotoxins have been discovered, most molds continue to exist in the environment. Contamination of feed during storage and processing cannot be completely avoided [11]. A large number of studies have shown that dairy cattle feed is often contaminated by multiple mycotoxins simultaneously, resulting in a synergistic risk [12,13,14]. This highlights the importance of assessing feed safety. Previous studies have reported that the combined contamination of ZEA and vomitoxin can affect the health status of breeding cattle [15]. Furthermore, ZEA has been shown to potentiate the toxicity of aflatoxin B1 in the rat liver and mammary gland [1]. Additionally, co-exposure to ZEA and fumonisin B1 exerts additive effects on zebrafish embryos, resulting in marked alterations in the immune and endocrine systems [16]. If ruminants consume contaminated feed and the toxins are not adequately degraded by rumen microbes, they may be transported through the circulatory system to target tissues such as the mammary gland [17]. At present, in addition to methods combining physics, chemistry and biology, plant extracts are also regarded as a feasible approach for removing mycotoxins.

Increasing evidence suggests that some antioxidants can effectively alleviate the toxicity caused by ZEA, including resveratrol [18], apigenin [19], curcumin [20], and rutin [21]. The above studies confirm the potential of antioxidants to alleviate the toxicity of ZEA. Sulforaphane (SFN) is a naturally occurring isothiocyanate organic sulfur compound that has been identified in cruciferous vegetables [22]. Consuming broccoli rich in SFN can enhance cellular antioxidant capacity, thereby protecting cells from oxidative stress. Studies have shown that SFN mediates oxidative stress and inflammatory responses by regulating key transcription factors [23,24,25]. Nuclear factor-erythroid 2-related factor 2 (Nrf2) plays a key role in preserving redox homeostasis by modulating the levels of antioxidant enzymes [26]. SFN acts in a Nrf2-dependent manner by activating antioxidant response elements, increasing the transcription of crucial antioxidant enzymes, including heme oxygenase-1, NAD(P)H:quinone oxidoreductase 1 (NQO-1), and glutathione S-transferase (GST) [27].

Studies have shown that SFN alleviates the toxicity of ZEA on porcine endometrial stromal cells by regulating mitochondrial function [28]. However, it is still unclear whether it has a similar protective effect on bovine mammary epithelial cells, and whether this is achieved through the activation of Nrf2. As is well known, the function of mammary epithelial cells directly affects lactation performance. This study aims to investigate the mitigating effects of SFN on oxidative damage in bovine mammary epithelial cells caused by ZEA. MAC-T cells were selected as an in vitro model. In the future, this may provide a theoretical basis for SFN as a potential nutritional additive to improve the health of the bovine mammary gland.

## 2. Materials and Methods

### 2.1. Chemicals and Reagents

Zearalenone (purity ≥ 99%) was purchased from Selleck Chemicals (S5676, Houston, TX, USA). Sulforaphane (HY-13755, purity ≥ 98%) and the Nrf2 inhibitor ML385 (Cat. No. HY-100523) was obtained from MedChemExpress (South Brunswick Township, NJ, USA). DMEM basal medium (6124323), fetal bovine serum (FBS, A5669701), and penicillin-streptomycin solution (15140122) were purchased from Gibco (Waltham, MA, USA). The reactive oxygen species (ROS) detection kit (R252), cell apoptosis detection kit (AD10), and mitochondrial membrane potential detection kit (MT09) were purchased from Dojindo Chemical Laboratories (Kumamoto, Japan). The SOD (A001-3-2), lactate dehydrogenase (LDH, A020-2-2), GSH (A006-2-1), and MDA (A003-4-1) assay kits were purchased from Nanjing Jiancheng Bioengineering Institute (Nanjing, China). The reverse transcription reagent (RR092A) and the fluorescent quantitative PCR reagent (CN830A) were both purchased from Takara Bio Inc. (Beijing, China). The BCA protein assay kit (23227), TRIzol reagent (15596026), and PVDF membranes (Immobilon^®^-P) were purchased from Merck KGaA (Darmstadt, Germany). The primary antibodies used in the experiment included Nrf2 (ab137550, 1:1000, Abcam, Cambridge, MA, USA), HO-1 (10701-1-AP, 1:1000), NQO1 (11451-1-AP, 1:1000), GCLM (14241-1-AP, 1:2000), and β-actin (20536-1-AP, 1:5000), all purchased from Proteintech (Wuhan, China). The HRP-conjugated goat anti-rabbit IgG secondary antibody (SA00001-2, 1:5000) was also purchased from Proteintech (Wuhan, China).

### 2.2. Cell Culture

MAC-T cells were obtained from Huzhen Industrial Co., Ltd. (Shanghai, China). MAC-T cells are cultured in vitro in high-glucose DMEM medium containing 10% fetal bovine serum, 1% penicillin-streptomycin, 1 μg/mL hydrocortisone, and 5 μg/mL insulin, and incubated at 37 °C with 5% CO_2_. ZEA and SFN were dissolved separately in dimethyl sulfoxide (DMSO). The stock solution was prepared to a final concentration of 100 mmol/L. The Nrf2 signaling pathway specific inhibitor ML385 was also dissolved in DMSO to prepare a 10 mmol/L stock solution. All the stock solutions were aliquoted and stored at −20 °C in the dark for future use.

### 2.3. Cell Viability Assay

After the cells were cultured, different concentrations of ZEA and SFN were applied to each group of cells separately, with a treatment duration of 24 h. It should be specially noted that, in the combined treatment group, ZEA (0, 5, 10, 20, 40, 60, 80, and 100 μM) and SFN (0, 1, 2.5, 5, 7.5 and 10 μM) were added to the culture medium simultaneously. After all treatment steps were completed, 10 µL of CCK-8 working solution was added to each well (37 °C, 1.5 h). The OD values at 450 nm of each well were read using a microplate reader, and the relative cell viability was calculated based on the values of the blank control group.

### 2.4. Lactate Dehydrogenase Assay

After completing cell culture, add different concentrations of ZEA (0, 5, 10, 20, 40 µM) and SFN (0, 1, 2.5, 5 µM) in 6-well plates, as well as combination treatments of ZEA and SFN (10 µM ZEA; ZEA 1 µM SFN; ZEA 2.5 µM SFN; ZEA 5 µM SFN). After 24 h, collect the cell culture medium to detect lactate dehydrogenase levels. Centrifuge the cell culture medium (4000 rpm, 5 min). Collect the supernatant for testing. Set up blank wells, test wells, standard wells, and control wells, add reagents in order, and gently shake to mix. Keep at room temperature for 5 min. Measure the absorbance at 440 nm using an enzymatic detector.

### 2.5. Reactive Oxygen Detection

The cells were cultured in 6-well plates. ZEA and SFN were added (control group; 10 µM ZEA group; ZEA + 1 µM SFN group; ZEA + 2.5 µM SFN group; ZEA + 5 µM SFN group). After cultivation, the liquid was discarded, and the cells were washed twice with 1 × PBS buffer. The fluorescent probe DCFH-DA was diluted with serum-free medium (1:1000). An amount of 1 mL of dye was added to each well. Incubate at 37 °C in the dark for 20 min. After the incubation is finished, discard the liquid and wash the cells three times with 1 × PBS buffer. The images were collected by a fluorescence microscope. Each experiment was independently repeated three times. For each repetition, three sample wells were set up, and at least three non-overlapping fields of view were randomly collected from each well. The fluorescence intensity of each field was measured using ImageJ software (V 1.54g). The average fluorescence intensity was calculated after background fluorescence correction to reflect the intracellular ROS level.

### 2.6. Mitochondrial Membrane Potential Detection

The cells were inoculated at an appropriate density in 6-well plates and cultured until a cell fusion degree of 70–80%. ZEA and SFN were used for treatment. After cultivation, remove the culture medium and wash twice with 1 × PBS. Add 1 mL of complete medium, followed by 1 mL of JC-1 working solution (the JC-1 probe and the buffer were diluted at a ratio of 1:100). After gentle mixing, place the cell culture plate in a 37 °C cell incubator and incubate it in the dark for 20 min. After cultivation is complete, discard the supernatant. Wash the cells twice with JC-1 staining buffer. Finally, 2 mL of 1 × PBS buffer was added to each well, and the images were observed and collected using a laser confocal microscope. JC-1 exists as a monomer in the cytoplasm (green fluorescence) and can form polymers when the mitochondrial membrane potential is high (red fluorescence). Each experiment was independently repeated three times, with three sample wells set for each repetition, and at least three non-overlapping fields were randomly collected from each well. The fluorescence intensity of each field was measured using ImageJ software, and the background fluorescence was subtracted before calculating the ratio.

### 2.7. GSH, SOD, and MDA Detection

The changes in MAC-T cells were detected using MDA, SOD and GSH kits. The sample processing procedure was as follows: discard the supernatant of the treated cells and scrape the cells directly using a cell scraper. Use the BCA kit to measure cellular protein. Add the reagents in sequence. GSH is measured by absorbance at 420 nm. SOD is measured by absorbance at 450 nm. MDA is measured by absorbance at 532 nm.

### 2.8. L-6, TNF-α, and IL-1β Detection

Collect the processed cells. Use the cell pellet for ELISA detection. Follow the instructions of the ELISA kit for operation: Add 50 μL of standard samples of each concentration, 50 μL of sample, and 50 μL of sample diluent. Then add 100 μL of HRP-labeled detection antibody to each well and seal the microplate (37 °C for 45 min). After finishing, discard the liquid. Add 350 μL of wash buffer, wait for 20 s, and then discard the liquid. Wash five times. After completion, add 50 μL of substrate solution A and B, and incubate in the dark at 37 °C for 15 min. Finally, add 50 μL of stop solution to terminate the reaction within 15 min. Use a microplate reader to detect the OD value at 450 nm.

### 2.9. Observation of Cell Structures by TEM

The cultured cells are digested with trypsin; after stopping the digestion, centrifuge and discard the supernatant, retaining the cell pellet. Prepare a diluted fixative by mixing 3% glutaraldehyde and 0.1 mol/L PBS buffer (1:1), slowly add it along the tube wall, and gently resuspend the cell pellet (4 °C for 5 min). Centrifuge and discard the supernatant (5000 rpm for 3 min), then slowly add 3% glutaraldehyde fixative. Store the fixed samples at 4 °C. Subsequently, postfix with 1% osmium tetroxide, perform graded acetone dehydration, infiltration and embedding. Finally, section and stain the samples. Image acquisition was carried out using the JEM-1400FLASH transmission electron microscope produced by JEOL (Tokyo, Japan).

### 2.10. Apoptosis Detection

Collect the processed cells and digest them with trypsin. Wash them twice with pre-cooled 1 × PBS—the cells were obtained through centrifugation. Prepare 1 × Binding Buffer. Add an appropriate amount of 1 × Binding Buffer to resuspend the cells and adjust the cell density to 1 × 10^6^ cells/mL. This suspension is aliquoted into flow cytometry tubes. Then, 5 μL of Annexin V-FITC and PI staining solution are added to each tube. The cell suspension is gently mixed and incubated at room temperature in the dark for 15 min. After the incubation, 400 μL of 1 × Binding Buffer is added to the tubes, gently mix, and immediately perform flow cytometry analysis on the machine. The proportions of early apoptosis and late apoptosis/necrosis cells were calculated using the software of the flow cytometer.

### 2.11. Quantitative Real-Time PCR

The purity of RNA was assessed by the A260/A280 ratio. All samples had measurements between 1.8 and 2.0, indicating good purity. Reverse transcription was performed according to the instructions from Takara, establishing a 20 µL system, divided into a two-step method. The first step is to add 2 µL (8 × gDNA Eraser Premix) and RNase-Free H_2_O to the RNA sample in a 200 µL centrifuge tube, totaling 16 µL (42 °C for 2 min). The second step is to add 4 µL (5 × RT Premix) to the 200 µL centrifuge tube, totaling 20 µL (37 °C for 10 min, 85 °C for 5 s, and finally cool to 4 °C). The synthesized cDNA should be stored at −20 °C or lower. For establishing a 20 µL fluorescent quantitative PCR system, 10 µL of TB Green Premix Ex Taq II Fast qPCR (2×) is required, 0.8 µL each of PCR Forward and Reverse Primers, 2 µL of cDNA, and 6.4 µL of sterile water, totaling 20 µL. The required temperatures and times are 95 °C for 30 s, 95 °C for 5 s, and 60 °C for 10 s, with 40 cycles. The primers were designed based on the genomic sequence of the cattle (*Bos taurus*) in the NCBI database. The relative expression levels of *Nrf2*, *NQO1*, *HO-1*, *GCLM*, *GPX1* and *β-actin* mRNA were calculated using the 2^−ΔΔCT^ method [29]. Table 1 shows the primer sequences.

### 2.12. Western Blot Analysis

After cell culture is completed, total cellular protein is extracted using RIPA lysis buffer. Determine the protein concentration. Mix the protein samples with loading buffer. Heat at 95 °C for 5 min to denature the proteins. Add 20 μg of protein sample to the wells of an SDS-PAGE gel and then transfer to a PVDF membrane. Incubate with the primary antibody overnight at 4 °C. After completing the three washes with 1 × TBST, react with HRP-conjugated Goat Anti-Rabbit IgG for 1 h at room temperature. The primary antibodies for HO-1, Nrf2, NQO1, GCLM, and GPX1 were obtained from Proteintech (Wuhan, China). Protein band densities were quantified using Image J software.

### 2.13. Immunofluorescence Assay

MAC-T Cells were seeded onto cell culture slides. After cultivation, the cells were washed twice with 1 × PBS solution and immediately fixed at room temperature with 4% paraformaldehyde for 15 min. After fixation, the permeability of the cells was treated with 0.5% Triton-X-100 for 15 min, followed by blocking with normal goat serum at room temperature for 30 min. Subsequently, the primary antibody against Nrf2 was added to the slides and left overnight at 4 °C. After incubation with the primary antibody, the fluorescent secondary antibody was introduced and co-incubated with the samples at 37 °C for 1 h. After all processing steps were completed, the slides were washed three times with PBS and mounted using DAPI mounting medium containing an anti-fade reagent. The final fluorescence images were all acquired using a laser confocal microscope.

### 2.14. Statistical Analysis

Based on experimental results from at least three independent biological replicates, all data are expressed as “mean ± standard deviation (SD)”. Statistical differences between different treatment groups were evaluated using one-way analysis of variance followed by Duncan’s test with SPSS 21.0 software. When *p* < 0.05, the result is considered statistically significant.

## 3. Results

### 3.1. Viability and Lactate Dehydrogenase of MAC-T Cells Affected by SFN and ZEA

**Figure 1:**
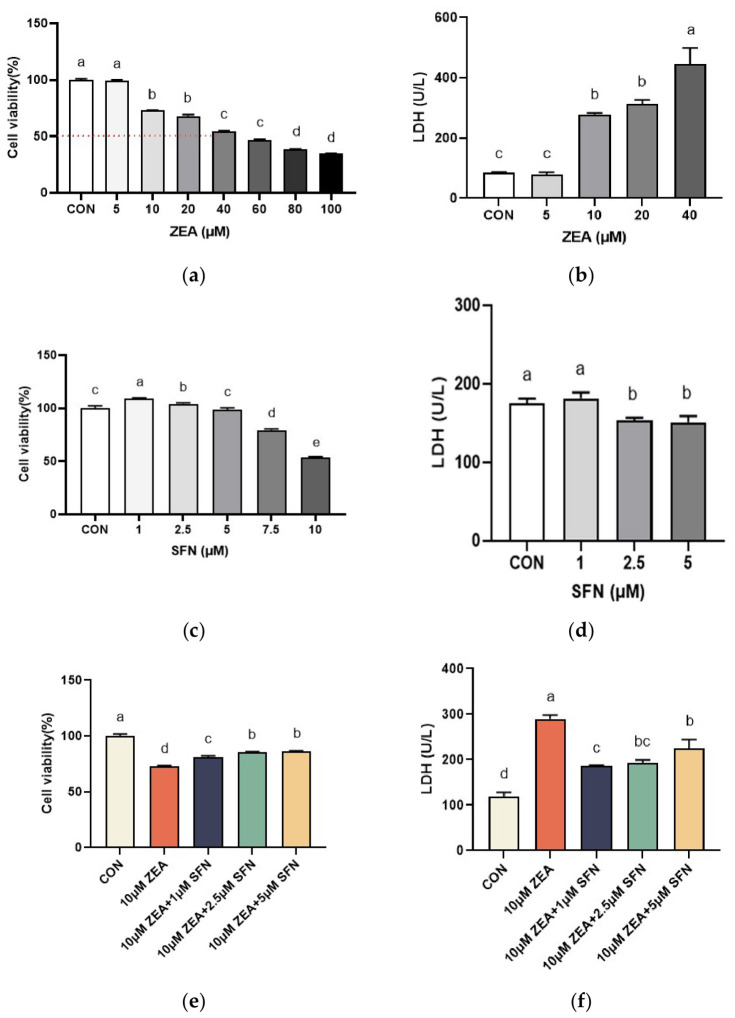
Changes in cell viability and lactate dehydrogenase in cells. (**a**) Cell viability after ZEA treatment. The red dashed line indicates the IC_50_ concentration of ZEA. (**b**) LDH after ZEA treatment. (**c**) Cell viability after SFN treatment. (**d**) LDH after SFN treatment. (**e**) Cell viability after treatment with 10 μM ZEA and 1, 2.5, and 5 μM SFN. (**f**) LDH after treatment with 10 μM ZEA and 1, 2.5, and 5 μM SFN. The above data are presented in the form of the mean ± SD of three independent experiments (*n* = 3). The letters a, b, c, d and e are indicated as *p* < 0.05.

Screen suitable doses of ZEA and SFN additives. First, after treating MAC-T cells with ZEA for 24 h, cell viability was measured. Compared with the control group (as shown in Figure 1a), treating cells with 5 μM ZEA had no significant effect on cell viability (*p* = 0.757), but treating with 10–100 μM ZEA significantly reduced cell viability (*p* < 0.05). The IC_50_ was approximately 42.69 μM. Compared with the control group, the cell viability in the 10 μM ZEA group decreased by 27.15%. Compared with the control group (as shown in Figure 1c), cell viability was significantly increased after treatment with 1 and 2.5 μM SFN (*p* < 0.05), unaffected by 5 μM SFN treatment (*p* = 0.517), but decreased after treatment with 7.5 and 10 μM SFN (*p* < 0.05). LDH results indicate (Figure 1b,d) that compared with the control group, LDH was significantly increased after treatment with 10–40 μM ZEA (*p* < 0.05). There was no difference after treatment with 1 μM SFN, but LDH levels decreased after treatment with 2.5 and 5 μM SFN (*p* < 0.05). As shown in Figure 1e,f, 10 μM ZEA and 1, 2.5, and 5 μM SFN were selected for subsequent combination studies. MAC-T cells treated with 10 μM ZEA show reduced cell viability and increased LDH levels (*p* < 0.05). After the addition of SFN, cell viability gradually increased, and LDH levels decreased (*p* < 0.05).

### 3.2. Antioxidant Capacity of MAC-T Cells Affected by SFN and ZEA

**Figure 2:**
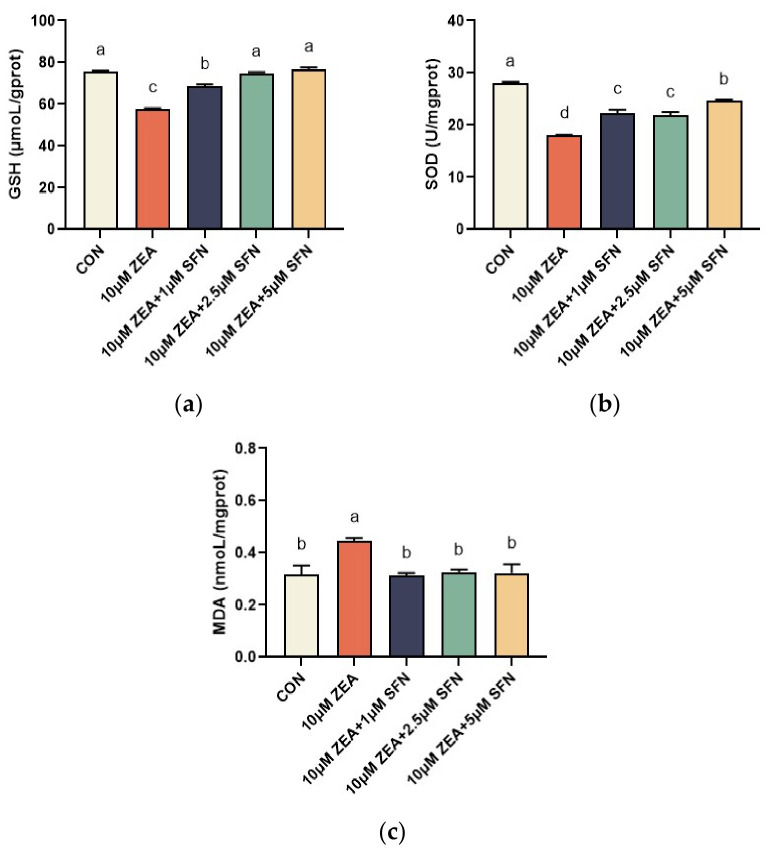
Determination of GSH, SOD and MDA in MAC-T cells. (**a**) GSH levels. (**b**) SOD levels. (**c**) MDA levels. Data from 3 independent experiments (*n* = 3) are presented as mean ± SD. The letters a, b, c and d are indicated as *p* < 0.05.

As shown in Figure 2a, the results indicate that after MAC-T cells were treated with ZEA, the GSH content was significantly reduced. The addition of SFN significantly increased the GSH content (*p* < 0.05). As shown in Figure 2b, the results of SOD detection indicate that SOD was significantly decreased in the 10 μM ZEA group, and SOD levels were significantly increased after the addition of SFN (*p* < 0.05), with the combination of 5 μM SFN and ZEA being more effective. As shown in Figure 2c, MDA detection results indicate that compared with the control group, MDA content significantly increased after ZEA treatment (*p* < 0.05). After the addition of SFN, MDA content was significantly reduced compared with the ZEA group (*p* < 0.05). This suggests that the cellular antioxidant defense system has been impaired, resulting in oxidative damage, while the addition of SFN effectively alleviates the damage.

### 3.3. Inflammatory Response and Apoptosis of MAC-T Cells Affected by SFN and ZEA

**Figure 3:**
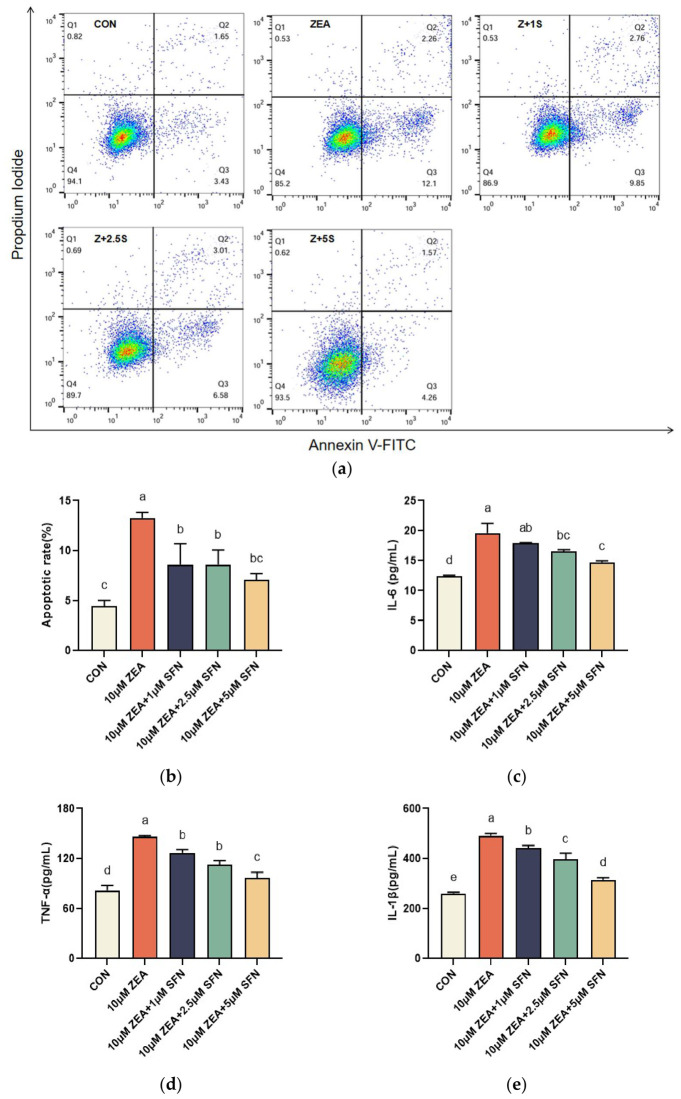
Changes in MAC-T cell apoptosis and inflammatory factors induced by SFN and ZEA. (**a**,**b**) Effects of SFN and ZEA on MAC-T cell apoptosis rates, with (**b**) plotted based on the apoptosis rates in (**a**). (**a**) Abbreviates the setup of CON, ZEA, ZEA (1, 2.5, or 5 μM) SFN. (**c**) IL-6 protein expression. (**d**) TNF-α protein expression. (**e**) IL-1β protein expression. Data from 3 independent experiments (*n* = 3) are presented as mean ± SD. The letters a, b, c, d and e are indicated as *p* < 0.05.

As shown in Figure 3a,b, cell apoptosis was detected by flow cytometry, and the apoptosis rate of cells treated with ZEA was significantly increased (*p* < 0.05), while it was significantly decreased when combined with 1, 2.5, and 5 μM SFN (*p* < 0.05). As shown in Figure 3c–e, compared with the control group, the IL-6, TNF-α, and IL-1β protein levels were significantly increased after ZEA treatment. (*p* < 0.05). Compared with the ZEA group, the IL-6 protein expression after the combination of 2.5 and 5 μM SFN with ZEA was significantly decreased (*p* < 0.05). At the same time, the TNF-α and IL-1β protein expressions after the combination of 1 and 2.5, and 5 μM SFN with ZEA were also significantly decreased (*p* < 0.05). Flow cytometry and ELISA analyses together revealed that ZEA induces oxidative stress, leading to increased cell apoptosis and release of inflammatory factors, whereas SFN, with its antioxidant and anti-inflammatory properties, successfully counteracted this process and restored cellular homeostasis.

### 3.4. Effects of SFN and ZEA on Reactive Oxygen Species, Mitochondrial Membrane Potential, and Ultrastructure of MAC-T Cells

**Figure 4:**
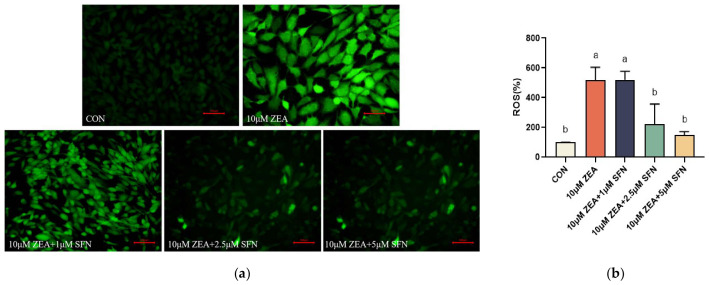

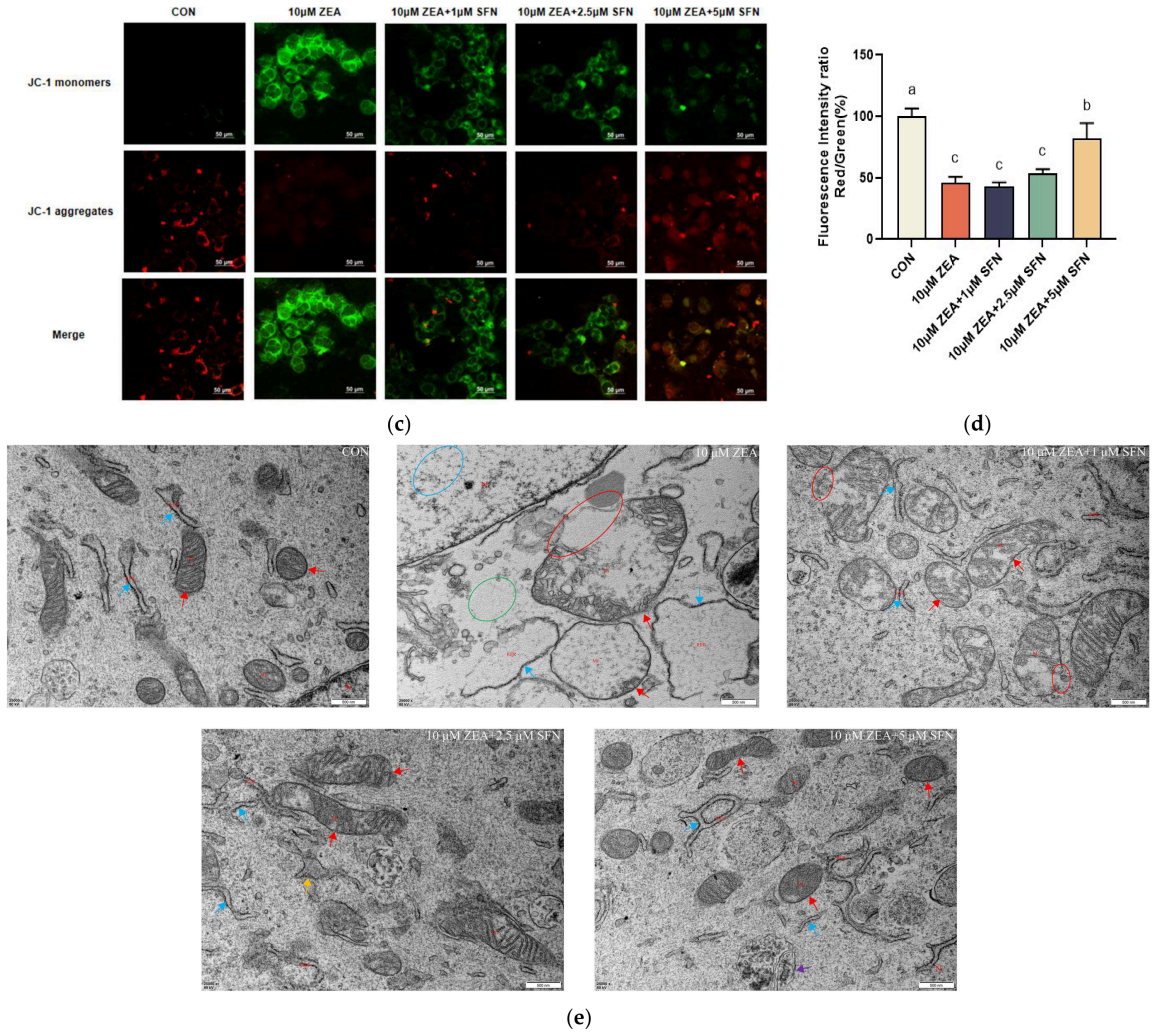
The levels of ROS, mitochondrial membrane potential, and cell structure were altered in MAC-T cells. (**a**) Observation of the effects of SFN and ZEA treatments on ROS levels in MAC-T cells under fluorescence microscopy (scale = 100 μm). (**b**) Quantitative analysis of the fluorescence intensity of ROS in each group. (**c**) Observation of the effects of SFN and ZEA treatments on mitochondrial membrane potential in MAC-T cells (scale = 50 μm). (**d**) The fluorescence intensity of the mitochondrial membrane potential was quantitatively analyzed. (**e**) Changes in the ultrastructure of MAC-T cells caused by SFN and ZEA treatment (scale = 500 nm). CON group: red arrows indicate normal-shaped mitochondria; N: cell nucleus; blue arrows indicate structurally normal rough endoplasmic reticulum (RER). The 10 µM ZEA group: red arrows indicate significantly swollen mitochondria; N: cell nucleus; blue arrows indicate dilated RER; blue circles indicate chromatin dissolution; green circles indicate cytoplasmic dissolution; red circles indicate rupture of the outer mitochondrial membrane. ZEA + 1 µM SFN group: red arrows indicate swollen mitochondria; blue arrows indicate structurally relatively normal RER; red circles indicate rupture of the outer mitochondrial membrane. ZEA + 2.5 µM SFN group: red arrows indicate slightly swollen mitochondria; blue arrows indicate structurally normal RER; yellow arrows indicate mild expansion of the rough endoplasmic reticulum. ZEA + 5 µM SFN group: red arrows indicate that the mitochondrial morphology tends to be normal; blue arrows indicate structurally normal RER; purple arrows indicate autophagic lysosomes. All experiments were independently repeated three times. For each repetition, 3 sample wells were set up for each group. At least 3 fields of view were randomly selected from each well for image acquisition and analysis to ensure the statistical reliability of the data (*n* = 3). The letters a, b, and c are indicated as *p* < 0.05.

Given that a typical feature of oxidative stress is the excessive production of ROS, this study further measured intracellular ROS levels. As shown in Figure 4a,b, the results indicated that, compared with the control group, cells treated with ZEA showed a significant increase in ROS (*p* < 0.05), and SFN at 2.5 and 5 μM was found to be more effective in reducing ROS (*p* < 0.05). Considering that mitochondria are both the main source of ROS within cells and a key target of ROS action, this study subsequently examined the mitochondrial membrane potential. The results showed that, as illustrated in Figure 4c,d, compared with the control group, the mitochondrial membrane potential was significantly reduced after ZEA treatment, and 5 μM SFN was found to increase mitochondrial membrane potential (*p* < 0.05). As shown in Figure 4e, the mitochondrial morphology and structure in the cells of the CON group were relatively normal. The chromatin was evenly distributed, mainly consisting of lightly colored euchromatin. The mitochondrial morphology and structure in the cytoplasm were also normal, and the rough endoplasmic reticulum (RER) morphology was normal. ZEA treatment causes the chromatin in the nucleus to dissolve, as well as the cytoplasm. The mitochondria in the cytoplasm were significantly swollen, and the RER was expanded. In the ZEA + 1 μM SFN group, the cell nucleus was irregular, the chromatin was evenly distributed, the mitochondria in the cytoplasm were swollen, and the RER morphology was normal or slightly expanded. In the ZEA + 2.5 μM SFN group, the cell nucleus was irregular, the mitochondria in the cytoplasm were slightly swollen, and the RER morphology was normal or slightly expanded. In the ZEA + 5 μM SFN group, the cell nucleus was nearly oval-shaped, the mitochondria in the cytoplasm were closer to normal or near normal, and the RER morphology was normal. A small amount of autophagic lysosomes could be seen in the cytoplasm of some cells.

### 3.5. Effects of SFN and ZEA on the Nrf2 Signaling Pathway in MAC-T Cells

**Figure 5:**
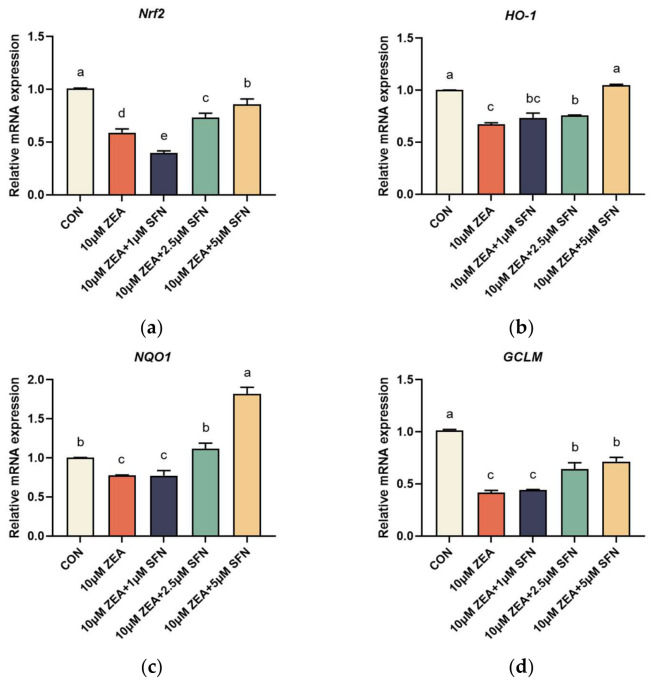

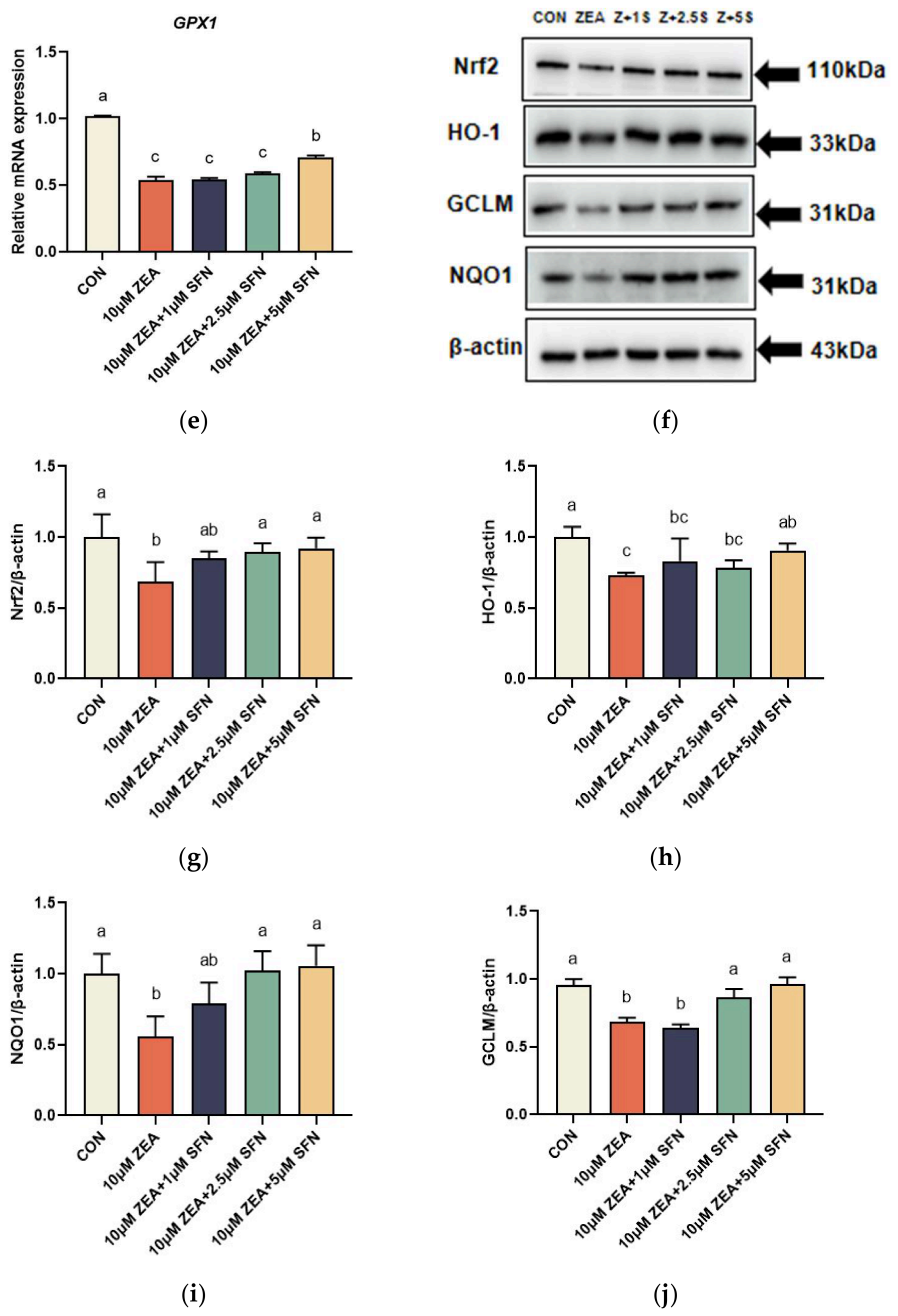
Effects of SFN and ZEA exposure on Nrf2 signaling pathway-related gene and protein expression in MAC-T cells. (**a**) *Nrf2* gene expression. (**b**) *HO-1* gene expression. (**c**) *NQO1* gene expression. (**d**) *GCLM* gene expression. (**e**) *GPX1* gene expression. (**f**–**j**) The protein expressions of SFN and ZEA were detected. Set up the control group (CON), 10 μM ZEA group (ZEA), 10 μM ZEA + 1 μM SFN group (Z + 1S), 10 μM ZEA + 2.5 μM SFN group (Z + 2.5S), and 10 μM ZEA + 5 μM SFN group (Z + 5S). Data from 3 independent experiments (*n* = 3) are presented as mean ± SD. The letters a, b, c, d and e are indicated as *p* < 0.05.

Since Nrf2 is an upstream key signaling pathway that regulates the antioxidant enzyme system and mitochondrial function, to elucidate the molecular mechanism of the above phenotype, we further examined the activation status of the Nrf2 signaling pathway. As shown in Figure 5a–e, compared with the control group, the expression of Nrf2-related genes in the ZEA group was significantly reduced (*p* < 0.05). In comparison with the ZEA group, treatment with 2.5 μM and 5 μM SFN significantly upregulated the expression of Nrf2, HO-1, NQO1, and GCLM; the significant increase in GPX1 expression was observed only under the combined treatment with 5 μM SFN (*p* < 0.05). As shown in Figure 5f–j, the results at the protein level were similar to those at the gene level. Compared with the control group, the ZEA group significantly reduced the expression of related protein levels (*p* < 0.05). In comparison with the ZEA group, treatment with 2.5 μM and 5 μM SFN significantly upregulated the expression of Nrf2, NQO1, and GCLM (*p* < 0.05); the significant increase in HO-1 expression was observed only under the combined treatment with 5 μM SFN (*p* < 0.05).

### 3.6. Effects of SFN and ZEA on the Expression and Nuclear Translocation of Nrf2 Protein

**Figure 6:**
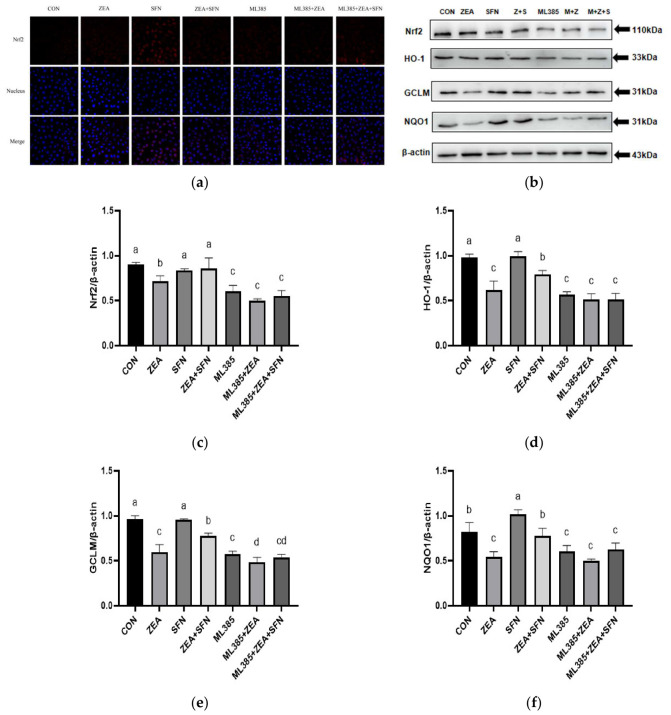
ML385 inhibitor suppresses SFN-induced Nrf2 protein expression and nuclear translocation in ZEA-treated MAC-T cells. (**a**) Immunofluorescence staining was used to detect Nrf2 (in red), the cell nucleus (DAPI, in blue), and the Merge image was the superimposition of the two (scale = 25 μm). (**b**–**f**) Relative expression of Nrf2, HO-1, NQO1, and GCLM proteins with ML385 inhibitor. ML385 (2 μM) was pre-treated for 1 h. The groups were set as: control group (CON), 10 μM ZEA group (ZEA), 5 μM SFN group (SFN), 10 μM ZEA + 5 μM SFN group (ZEA + SFN), 2 μM ML385 group (ML385), 2 μM ML385 + 10 μM ZEA group (ML385 + ZEA), and 2 μM ML385 + 10 μM ZEA + 5 μM SFN group (ML385 + ZEA + SFN). Data from 3 independent experiments (*n* = 3) are presented as mean ± SD. The letters a, b, c and d are indicated as *p* < 0.05.

This study used the Nrf2-specific inhibitor ML385 for validation. As shown in Figure 6a, in the SFN group and the ZEA + SFN combined treatment group, Nrf2 was significantly concentrated in the cell nucleus and co-localized significantly with the DAPI staining of the cell nucleus. However, ML385 significantly reduced the accumulation of Nrf2 in the cell nucleus caused by SFN. As shown in Figure 6b–f, the protein levels in the ZEA group and the ML385 group were significantly reduced compared with the control group (*p* < 0.05). The ZEA group was similar to the ML385 + ZEA + SFN group. The protein levels in both groups were significantly lower than that in the ZEA + SFN group (*p* < 0.05). This further confirms that SFN can alleviate cell oxidative stress caused by ZEA.

## 4. Discussion

ZEA mainly affects those tissues with high expression of estrogen receptors (ES), including the ovaries, uterus, mammary glands, testes, etc. [30]. The susceptibility of breast tissue to ZEA may be related to its abundant blood circulation, active metabolic state, and high expression of estrogen receptors. These factors jointly contribute to the potential risk of ZEA accumulation in the breast. Studies on other types of cells have shown that ZEA has a dose-dependent effect on the activity of sheep follicular granulosa cells, and ZEA inhibits the proliferation and survival capacity of trophoblast ectoderm (pTr) cells [31]. Similarly, our study indicates that 10–100 μM ZEA inhibits MAC-T cell viability. Mitochondria, as the primary location of ROS production and their goal within cells, not only provide energy for cells but also participate extensively in important biological processes such as cell differentiation, apoptosis, and signal transduction. Previous research has shown that ZEA can affect mitochondrial activity, number, structure, and function [32]. Mitochondrial damage induced by ZEA is likely a key pathway promoting the production of ROS [33]. Excessive ROS production can trigger cellular oxidative stress, subsequently leading to increased levels of lipid peroxidation products and decreased indicators related to antioxidant capacity [34]. Our research has shown that treating MAC-T cells with 10 μM ZEA significantly reduced the activity of the intracellular antioxidant enzyme SOD, the content of the antioxidant substance GSH, and the mitochondrial membrane potential. Observing the cell morphology and structure revealed that the mitochondria and endoplasmic reticulum of the cells treated with ZEA underwent changes. These findings indicate that ZEA treatment of MAC-T cells causes oxidative damage. Previous studies have also shown that 10 and 20 μM ZEA significantly reduce the cell viability of bovine mammary epithelial cells, induce oxidative stress, trigger programmed cell death, and impair milk fat synthesis [35]. Similar research results have shown that ZEA can induce cell apoptosis, and it also increases the levels of ROS and MDA in porcine endometrial stromal cells (ESCs), and significantly enhances cell viability, the activities of GSH-Px and T-SOD enzymes [36].

We discovered that ZEA induces cell apoptosis and increases the protein levels of IL-6, IL-1β and TNF-α. ZEA can induce apoptosis in IPEC-J2 cells and increase the expression of inflammatory-related genes [37]. ZEA induces an increase in the apoptosis rate and ROS levels of porcine endometrial epithelial cells (PEECs) [38]. Similar results were also observed in ZEA in LS174T cells [39]. These results suggest a close interrelationship between ZEA-induced cell apoptosis and inflammatory responses. Numerous studies have shown that cell apoptosis and inflammatory signaling pathways do not exist independently, but rather regulate each other through various molecular mechanisms [40]. For example, mitochondrial dysfunction can release mitochondrial DNA (mtDNA) and ROS, and these damage-associated molecular patterns (DAMPs) can activate the NLRP3 inflammasome, thereby promoting the maturation and secretion of inflammatory factors and exacerbating the inflammatory response [41,42]. Therefore, in ZEA-induced cytotoxicity models, apoptosis and inflammation may form a vicious cycle: initial cell damage triggers the release of inflammatory factors, and the inflammatory microenvironment further promotes cell apoptosis. This view is consistent with the previous literature. Among other mycotoxins, deoxynivalenol (DON) has been proven to trigger apoptosis and inflammatory responses [43,44]. In summary, the apoptosis and high expression of inflammatory factors caused by ZEA are not isolated events, but rather are mutually promoting and jointly involved in the process of cell damage.

Natural compounds in many foods are known to have antioxidant effects. SFN, an electrophilic compound abundant in cruciferous vegetables like broccoli, strongly activates cellular antioxidant defenses [45]. The results showed that, compared with treatment with ZEA alone, the combination of 1, 2.5, and 5 μM SFN with ZEA could increase cell viability, GSH, and SOD levels, and reduce LDH and MDA levels. At the same time, the combination of 2.5 and 5 μM SFN with ZEA could reduce ROS accumulation, and the combination of 5 μM SFN with ZEA increased mitochondrial membrane potential. SFN can effectively enhance the activity of SOD, increase the ratio of GSH to GSSG, and reduce the generation of ROS and MDA caused by H_2_O_2_ as well as the phenomenon of cell apoptosis, which has been studied and found [46].

During the early lactation stage, the mammary epithelial cells of cows undergo severe metabolic challenges and oxidative stress. SFN attenuates the oxidative stress induced by exogenous free fatty acids by activating Nrf2-mediated autophagy [47]. Nrf2 is a crucial transcription factor that plays a role in alleviating oxidative stress and maintaining cellular homeostasis [48]. Existing studies have shown that various bioactive compounds derived from natural foods and nutrients (such as curcumin, resveratrol, sulforaphane, and vitamin D) can enhance the antioxidant defense capacity by activating Nrf2 [49,50]. SFN has been confirmed as an efficient activator of the Nrf2 signaling pathway, capable of significantly inducing the expression of phase II detoxification enzymes and enhancing the detoxification and antioxidant capacity of cells [51,52]. ZEA can reduce the expression of Nrf2 and its downstream genes. SFN can significantly reverse this effect, which has been confirmed by this study. The inhibitor ML385 was utilized for verification. The results showed that ML385 inhibited the protective effect of ZEA exerted by SFN. The alleviation of oxidative damage induced by ZEA by SFN may be attributed to the activation of the Nrf2 signaling pathway. From the perspective of dairy production, the traditional mycotoxin removal strategies mainly target the toxins present in the feed. However, this study suggests that by using nutritional means, the endogenous antioxidant system of the animals can be stimulated, which may become an effective supplementary strategy.

This study confirmed that SFN alleviates ZEA-induced damage in MAC-T cells by activating the Nrf2 mediated antioxidant response. Given the difficulty of completely eliminating ZEA contamination in feed, SFN or plant extracts rich in SFN have potential as nutritional additives to reduce the potential risk of ZEA. Currently, this study is at the cellular level, and future in vivo experiments are needed to determine the appropriate supplementation scheme of SFN in the diet of dairy cows and to evaluate its effects on metabolism, bioavailability, and safety, in order to provide a basis for developing SFN as a nutritional additive.

## 5. Conclusions

The results of this study indicate that in MAC-T cells, SFN can activate the Nrf2 pathway and alleviate the oxidative stress caused by ZEA. Future research needs to further evaluate the activation effect of SFN on the Nrf2 pathway in dairy cow mammary tissue and its protective role in breast health under ZEA exposure.

## Figures and Tables

**Table 1:** Gene information and PCR primer sequences.

Genes	Forward/Reverse Primer (5′-3′)	GenBank Accession	Product Size (bp)
*β-actin*	F:CCATCGGCAATGAGCGGTTC	NM_173979.3	98
	R:GGAATTGAAGGTAGTTTCGTGAATGC		
*HO-1*	F:GCCAGTGCCACCAAGTTCAAG	NM_001014912.1	112
	R:TGAGCAGGAAGGCGGTCTTG		
*Nrf2*	F:TTTGGCAGAGACATTCCCGTTTG	NM_001011678.2	119
	R:CCTGAGGAGGAGCAGTGAAGAC		
*NQO1*	F:ATGAAGGAGGCTGCCATAGAGG	NM_001034535.1	95
	R:CTGGAGATGACGGGATTGAAGTTC		
*GCLM*	F:TCTTGCCTCCTGCTGTGTGATG	NM_001038143.1	136
	R:GATGCTCTCCTGAAGTGCTTCTTG		
*GPX1*	F:ATCCGCTCTTCGCCTTCCTTC	NM_174076.3	93
	R:GGGACCAGGTGATGAACTTAGGG		

## Data Availability

The original contributions presented in this study are included in the article. Further inquiries can be directed to the corresponding authors.

## Abbreviations

The following abbreviations are used in this manuscript:

ZEA	Zearalenone
SFN	Sulforaphane
LDH	Lactate dehydrogenase
ROS	Reactive oxygen species
GSH	Glutathione
SOD	Superoxide dismutase
MDA	Malondialdehyde
Nrf2	Nuclear factor erythroid 2–related factor 2
HO-1	Heme oxygenase 1
NQO1	NAD(P)H:quinone oxidoreductase 1
GCLM	Glutamate-cysteine ligase modifier subunit
Gpx1	Glutathione peroxidase 1
